# Risk Factors for Anemia Exacerbation Requiring Red Blood Cell Transfusion During Edoxaban Thromboprophylaxis After Orthopedic Surgery

**DOI:** 10.7759/cureus.64320

**Published:** 2024-07-11

**Authors:** Yasuhisa Izushi, Naofumi Shiota, Tomonori Tetsunaga, Yusuke Ookura, Toru Sato, Yoshihisa Kitamura

**Affiliations:** 1 Department of Pharmacotherapy, School of Pharmacy, Shujitsu University, Okayama, JPN; 2 Division of Molecular and Cellular Medicine, Department of Clinical Science, National Hospital Organization Okayama Medical Centre, Okayama, JPN; 3 Department of Orthopedic Surgery and Rehabilitation, National Hospital Organization Okayama Medical Centre, Okayama, JPN; 4 Department of Intelligent Orthopedic System, Faculty of Medicine, Dentistry and Pharmaceutical Sciences, Okayama University, Okayama, JPN; 5 Department of Pharmacy, National Hospital Organization Okayama Medical Centre, Okayama, JPN; 6 Department of Orthopedic Surgery, National Hospital Organization Okayama Medical Centre, Okayama, JPN

**Keywords:** coagulation parameters, risk factors, anemia, preoperative renal function, intraoperative blood loss volume, preoperative hemoglobin levels, orthopedic surgery, red blood cell transfusion, postoperative edoxaban thromboprophylaxis

## Abstract

Background

An increased risk of anemia during edoxaban thromboprophylaxis in orthopedic surgery has been reported. However, the risk factors for the exacerbation of anemia requiring transfusion with irradiated red blood cell concentrates during postoperative edoxaban thromboprophylaxis remain unknown. Therefore, this study investigated the factors that increase the possibility of transfusion during edoxaban thromboprophylaxis after orthopedic surgery by reanalyzing clinical data from a previous collection.

Methods

A total of 221 patients who underwent total hip or knee arthroplasty at a single center between July 2011 and June 2012 were included in this study. Among these, 167 patients who received 30 mg of postoperative edoxaban thromboprophylaxis were retrospectively analyzed to identify critical factors for transfusion during edoxaban thromboprophylaxis after orthopedic surgery.

Results

Lower preoperative hemoglobin levels and higher intraoperative blood loss per body weight were significantly associated with an increase in the frequency of transfusion during postoperative edoxaban thromboprophylaxis. These factors were also potentially related to increased coagulation parameters during edoxaban thromboprophylaxis.

Conclusion

Our study shows that lower preoperative hemoglobin levels and higher intraoperative blood loss are associated with increased coagulation parameters with edoxaban thromboprophylaxis after orthopedic surgery and may lead to worsening of anemia, thereby requiring blood transfusion.

## Introduction

Patients undergoing orthopedic surgery, such as total knee arthroplasty and total hip arthroplasty (TKA/THA), are exposed to a high risk of bleeding from surgical wounds [1]. However, anticoagulant thromboprophylaxis is recommended to prevent venous thromboembolism (VTE) [2,3].

Edoxaban is a direct Xa inhibitor that is widely prescribed in Japan to prevent VTE after orthopedic surgery for TKA/THA [4]. As a convenient anticoagulant, edoxaban is orally administered once daily at a fixed dosage without routine monitoring [4]. However, we have already reported that postoperative edoxaban thromboprophylaxis significantly increased the frequency of postoperative anemia compared to fondaparinux (the leading postoperative thromboprophylaxis agent used before edoxaban approval) in a case-control study [5,6]. This increase in postoperative anemia with edoxaban thromboprophylaxis was possibly related to a significant prolongation of prothrombin time (PT) and international normalized ratio of PT (PT-INR) with edoxaban treatment and an increase in the estimated postoperative blood loss with prolongation of these coagulation parameters. Particularly, patients with impaired preoperative renal function have an increased risk of postoperative anemia during postoperative edoxaban thromboprophylaxis [7]. Additionally, reports from these and other studies have shown that edoxaban thromboprophylaxis may tend to increase the number of cases requiring transfusion and total transfusion doses compared to the non-treated group [5-8].

However, the risk factors for the exacerbation of anemia requiring transfusion during postoperative edoxaban thromboprophylaxis remain unknown. Therefore, we investigated the critical factors responsible for increasing irradiated red blood cell (iRBC) concentrate frequency due to anemia during edoxaban thromboprophylaxis after orthopedic surgery by reanalyzing clinical data from a previous collection [7].

Portions of this study were presented as meeting abstracts at the 6th Kinki Regional Meeting of the Japanese Society of Clinical Pharmacology and Therapeutics held on July 16, 2022, and at the 15th Annual Meeting of the Japanese Society of Clinical Trials and Research held on March 8, 2024.

## Materials and methods

Ethics approval

This study was approved by the Institutional Review Board of the National Hospital Organization (NHO) at the Okayama Medical Centre (approval number: 2019-056). All procedures involving human participants were performed in accordance with the Ethical Guidelines for Medical and Health Research Involving Human Subjects in Japan and the principle of the Declaration of Helsinki. Since this study had a retrospective design, and anonymity was secured, the Institutional Review Board of the NHO at the Okayama Medical Centre waived the requirement for written informed consent, and an opt-out method was applied by notifications displayed on the hospital’s website.

Patient criteria

**Figure 1:**
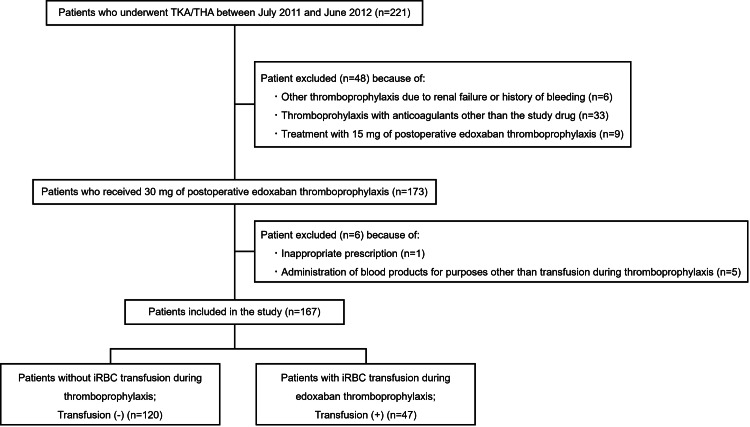
Diagram showing the details of patients included or excluded from the study TKA: total knee arthroplasty; THA: total hip arthroplasty

This retrospective observational study reanalyzed preexisting patient data [7]. All patient data were extracted from medical records and the anesthesia information management system under close supervision. Furthermore, the collected data were used only after the accuracy was verified by three medical experts. Overall, data from 221 patients aged ≥20 years who underwent TKA/THA at the NHO Department of Orthopedic Surgery between July 2011 and June 2012 were extracted. Among them, based on previous reports [7] and the exclusion criteria shown in Figure 1, 48 patients who had received prophylaxis with anticoagulants other than edoxaban or edoxaban at a reduced dose were excluded, and 173 patients who received anticoagulant thromboprophylaxis with oral administration of 30 mg of edoxaban once daily postoperatively were selected. Six patients who had received inappropriate prescriptions or blood products were excluded, and the remaining 167 patients were eligible for the study. The patients were categorized based on whether they received iRBC concentrate transfusions during edoxaban thromboprophylaxis. A total of 47 patients who received iRBC concentrate transfusions were eligible for enrollment in the case group (transfusion (+)), and 120 were eligible for enrollment in the control group (transfusion (-)) (Figure 1). The exclusion criteria are described in detail in a previous report [7].

Treatment

The treatment of patients after orthopedic surgery has been previously described in detail [7]. Briefly, 30 mg of edoxaban was orally administered once daily from 12 to 24 hours postoperatively for 14 days (Japanese standard of care). During edoxaban thromboprophylaxis, there were no cases in which the dose of edoxaban was changed from the standard dose of 30 mg. On the other hand, edoxaban thromboprophylaxis was discontinued in 28 patients. Of these, the number of patients who discontinued thromboprophylaxis for the bleeding-related event was significantly higher in the transfusion (+) group than in the transfusion (-) group, but not different for other reasons between these groups (Table 4 in Appendices). Blood tests were performed preoperatively and on postoperative days (PODs) one, three, seven, and 14 during edoxaban thromboprophylaxis. During this time, at least two units of iRBC concentrate transfusions were administered to patients whose hemoglobin (Hgb) levels had decreased to 7.5 g/dL or those determined by a doctor to have symptomatic anemia requiring iRBC transfusion. All data for patients treated with edoxaban were collected from the preoperative assessment until POD 14 or treatment discontinuation. Blood coagulation assays, including PT, INR of prothrombin time (PT-INR), and activated partial thromboplastin time (APTT), were conducted, as previously described [5]. These coagulation parameters and Hgb levels at POD3 were used for assessment in this study because the coagulation parameters were the longest, and Hgb levels were lowest on POD3 during edoxaban thromboprophylaxis [6,7].

During the study period, standardized surgical methods and procedures for anesthesia were used for patients undergoing TKA/THA. The patient’s treatment with the orthopedic procedure during the perioperative period in each surgery has been previously described in detail [7]. Mechanical thromboprophylaxis, including intermittent pneumatic compression therapy, elastic stockings, early postoperative ambulation, and physical rehabilitation, was performed in all patients along with edoxaban thromboprophylaxis to prevent the onset of deep vein thrombosis.

Assessment of estimated blood loss and clinically relevant bleeding-related events during edoxaban thromboprophylaxis

The estimated blood loss volume per body weight (eBLV) after edoxaban thromboprophylaxis initiation was analyzed by calculating the circulating blood loss using the Hgb balance calculation method [6,9]. First, the amount of circulating blood was calculated using the following formula [1]; the volume of each patient’s circulating blood (L) = (k1*height [m]) + (k2*body weight [kg]) + k3 (k1=0.3669, k2=0.03219, and k3=0.6041 for men and k1=0.3561, k2=0.03308, and k3=0.1833 for women). These ‘k’ values are constants of calculating formulas that were investigated for the estimation of accurate circulation blood volume of normal adult humans [10]. Next, the loss of postoperative Hgb during edoxaban thromboprophylaxis was estimated as follows [11]: Hgb loss (g) = patient’s blood volume (L) × 10*(ΔHgb) + Hgb_t_; ΔHgb was defined as the decrease in Hgb values from POD1 to POD3, the lowest Hgb value during edoxaban thromboprophylaxis. Hgb_t_ (g) is the amount of Hgb supplemented by iRBC. Finally, the total eBLV was calculated as follows [9]: eBLV (mL) = 100 × Hgb loss (g) / Hgb_pre_. Hgb_pre_ (g/dL) is the preoperative Hgb level.

Clinically relevant bleeding-related events were defined at the physician’s discretion, leading to the decision to discontinue edoxaban treatment. The degree of anemia was determined based on the lowest Hgb level during edoxaban thromboprophylaxis.

Statistical analyses

Continuous variables are expressed as means ± standard deviations. Comparisons were performed to explore the underlying risk factors that differed between the groups using the Mann-Whitney U test for continuous variables or Fisher’s exact test for categorical variables. Additionally, univariate logistic regression analyses were performed to select significant factors for use in multivariate logistic regression analysis. Multivariate logistic regression analysis was used to obtain the odds ratios (ORs) for the factors exhibiting the greatest ORs in the univariate logistic regression analyses to identify the risk factors for the exacerbation of anemia. Multicollinearity was assessed using the variance inflation factor (VIF). The VIF values did not exceed 10, and the mean VIF was <3 in this analysis. Evidence of multicollinearity indicates inconsequential collinearity [12]. For each risk factor that exhibited significance in multivariate logistic regression analysis, the optimal cutoff point of the risk factor for anemia was calculated to optimize the sensitivity and specificity. Receiver operating characteristic (ROC) curves were plotted, and the areas under the ROC curve (AUC) were calculated to determine the optimal threshold for classification accuracy. The Delong test was performed to compare the significant difference between the two AUCs of the ROC curve. Correlations between continuous variables and investigated factors were evaluated using Spearman's correlation coefficients. Continuous variables were also analyzed using analysis of variance (ANOVA), followed by Tukey’s multiple comparison test. Statistical significance was set at P<0.05. All statistical analyses were performed using GraphPad Prism 10.2.2 software (GraphPad Software Inc., San Diego, CA).

## Results

Differences in background medical factors with and without blood transfusion during edoxaban thromboprophylaxis

Table 1 presents the preoperative characteristics of the patients in the groups with or without transfusion during edoxaban thromboprophylaxis. The eight significantly different factors between the groups with and without transfusion were age, height, body weight, creatinine clearance (CrCl), Hgb, intraoperative blood loss volume per body weight (iBLV), PT, and PT-INR. On the other hand, there were no significant differences in combined medications that may potentiate the effects of edoxaban, such as P-glycoprotein inhibitors and anti-thrombogenic drugs, between the two groups.

**Table 1:** Characteristics of patients in the groups with or without transfusion at preoperative assessment Data are expressed as the mean±standard deviation or absolute value. #Patients were excluded if data were missing. TKA: total knee arthroplasty; THA: total hip arthroplasty; CrCl: creatinine clearance; Hgb: hemoglobin; iBLV: intraoperative blood loss volume per body weight; PT: prothrombin time; PT-INR: prothrombin time-international normalized ratio; APTT: activated partial thromboplastin time

Characteristics	Transfusion (-) N=120	Transfusion (+) N=47	P-value
Sex (male/female)	23 / 97	8 / 39	0.828
Age (years)	71.9 ± 9.1	76.4 ± 7.9	0.002
Surgical method (TKA/THA)	76 / 44	32 / 15	0.594
Height (cm)	153.1 ± 7.9 (n=117^#^)	150.0 ± 8.2 (n=44^#^)	0.022
Body weight (kg)	61.6 ± 12.0 (n=117^#^)	53.3 ± 9.8 (n=44^#^)	<0.001
CrCl (mL/min)	78.7 ± 29.7 (n=117^#^)	60.4 ± 18.2 (n=44^#^)	<0.001
Hgb (g/dL)	13.1 ± 1.4	11.7 ± 1.4	<0.001
iBLV (mL/kg)	3.35 ± 2.31 (n=108^#^)	4.79 ± 3.56 (n=39^#^)	0.020
PT	13.2 ± 1.1	13.4 ± 0.6	0.002
PT-INR	1.01 ± 0.11	1.03 ± 0.07	0.004
APTT	35.3 ± 3.4	35.9 ± 4.1	0.514
Combined anti-thrombogenic drug use (+/-)	21 / 99	10 / 37	0.659
Combined P-glycoprotein inhibitor use (+/-)	3 / 117	3 / 44	0.352

Univariable and multivariable logistic regression analysis of the risk factors for transfusion during edoxaban thromboprophylaxis

Univariate and multivariate logistic regression analyses were performed to identify the most important risk factors for transfusion in patients who received postoperative edoxaban thromboprophylaxis (Table 2). After univariate logistic regression analysis, six independent risk factors, i.e., age, height, body weight, CrCl, Hgb, and iBLV, were found to be associated with transfusion (Table 2). Finally, iBLV and Hgb levels were significantly associated with an increase in transfusion frequency during postoperative edoxaban thromboprophylaxis (OR: 2.139, 95% confidence interval (CI): 1.505-3.228, VIF: 1.220, P<0.001; OR: 0.781, 95% CI: 0.657-0.915, VIF: 1.014, P=0.003, respectively).

**Table 2:** Univariable and multivariable logistic regression analyses of risk factors for transfusion during edoxaban treatment OR: odds ratio; CI: confidence interval; VIF: variance inflation factor; CrCl: creatinine clearance; Hgb: hemoglobin; iBLV: intraoperative blood loss volume per body weight; PT: prothrombin time; PT-INR: prothrombin time-international normalized ratio

	Univariable logistic regression analyses			Multivariable logistic regression analysis			
Factors	OR	95% CI	P-value	OR	95% CI	VIF	P-value
Age	1.070	1.024–1.124	0.005	0.948	0.865–1.034	2.990	0.238
Height	0.949	0.903–0.994	0.031	0.977	0.901–1.056	1.872	0.564
Body weight	0.932	0.896–0.965	<0.001	1.030	0.969–1.097	2.649	0.349
CrCl	0.969	0.950–0.985	<0.001	1.006	0.975–1.039	3.845	0.711
Hgb	0.503	0.370–0.659	<0.001	2.139	1.505–3.228	1.220	<0.001
iBLV	1.192	1.049–1.365	0.008	0.781	0.657–0.915	1.014	0.003
PT	1.235	0.885–1.765	0.210				
PT-INR	7.026	0.276–216.1	0.231				

Calculation of cutoff values for each risk factor using ROC curve analysis and comparison of the AUC

ROC curve analysis was used to confirm the potential of Hgb levels and iBLV as predictors of transfusion during postoperative edoxaban thromboprophylaxis. Consequently, the cutoff values for Hgb level and iBLV were calculated as 12.25 g/dL (AUC: 0.766 (95% CI: 0.688-0.845), sensitivity: 68.1%, specificity: 75.8%, P<0.001) and 2.59 mL/kg (AUC: 0.626 (95% CI: 0.523-0.729), sensitivity: 71.8%, specificity: 49.1%, P=0.020), respectively (Table 3). Additionally, the AUC of Hgb level was significantly higher than that of iBLV.

**Table 3:** Area under the ROC curve of intraoperative bleeding volume and preoperative Hgb levels as predictors of the frequency of red blood cell transfusion during anticoagulant thromboprophylaxis ROC: receiver operating characteristic; CI: confidence interval; AUC: area under the ROC curve; Hgb: hemoglobin; iBLV: intraoperative blood loss volume per body weight

Factors	Cutoff value	AUC（95% CI）	Sensitivity (%)	Specificity (%)	P-value
Hgb	12.25 g/dL	0.766 (0.688-0.845)	68.1	75.8	0.031
iBLV	2.59 mL/kg	0.626 (0.523-0.729)	71.8	49.1	

Effect of preoperative Hgb level and iBLV on the frequency of transfusion and bleeding-related events during edoxaban thromboprophylaxis

**Figure 2:**
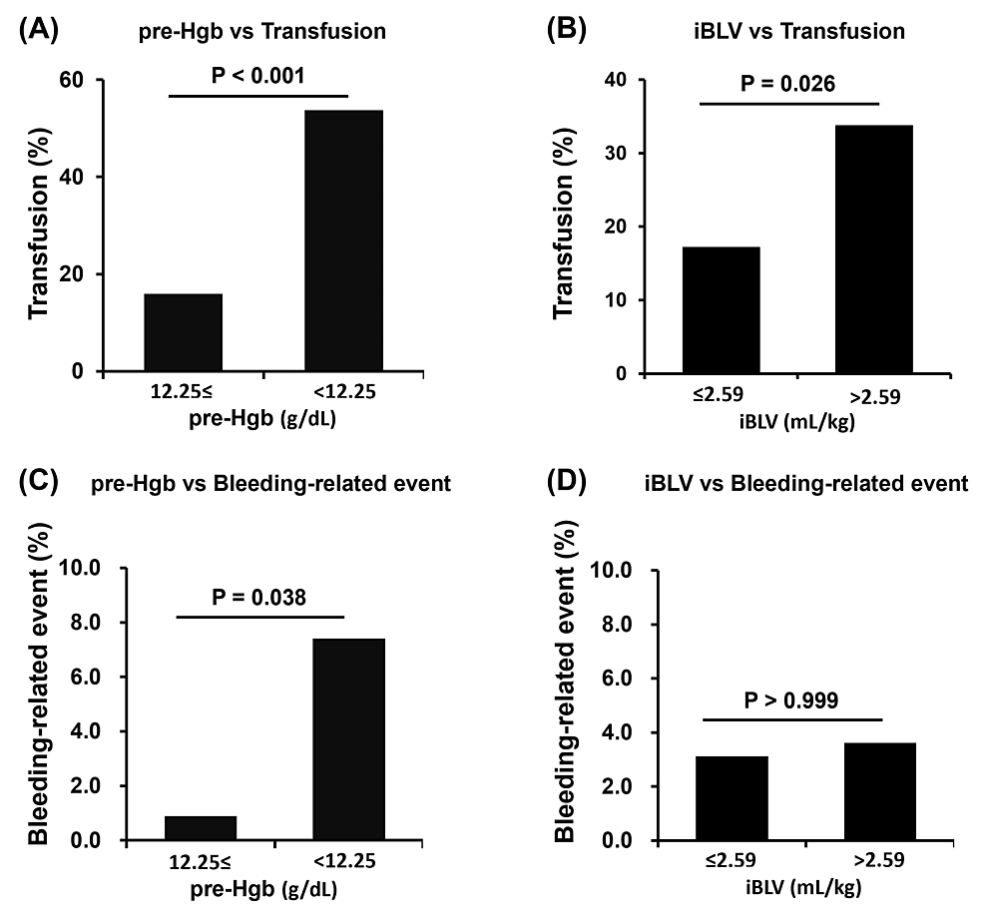
Association between preoperative anemia or increased intraoperative blood loss and clinically relevant bleeding and iRBC transfusion Preoperative anemia and increased intraoperative blood loss increase the frequency of blood transfusions during edoxaban thromboprophylaxis. Additionally, preoperative anemia significantly increases the incidence of bleeding-related events. Significant differences were determined using Fisher’s exact test. The P-values are shown in each figure. Pre-Hgb: preoperative hemoglobin; iRBC: irradiated red blood cell; iBLV: intraoperative blood loss volume per body weight

To evaluate the effect of preoperative Hgb levels or iBLV on the frequency of transfusion and bleeding-related events during edoxaban thromboprophylaxis, the patients were categorized into two groups based on the cutoff value for each factor, and these groups were compared in terms of the frequency of transfusion and bleeding-related events (Figure 2). The frequency of transfusion was significantly higher in patients with lower Hgb levels or an increased iBLV (Figures 2A, 2B). The frequency of bleeding-related events was significantly higher in patients with lower Hgb levels (Figure 2C). However, iBLV was not related to the frequency of bleeding-related events (Figure 2D). These results indicate that lower preoperative Hgb levels may be associated with clinical blood loss during edoxaban thromboprophylaxis.

Correlation of preoperative Hgb levels and iBLV with postoperative estimated blood loss during edoxaban thromboprophylaxis

**Figure 3:**
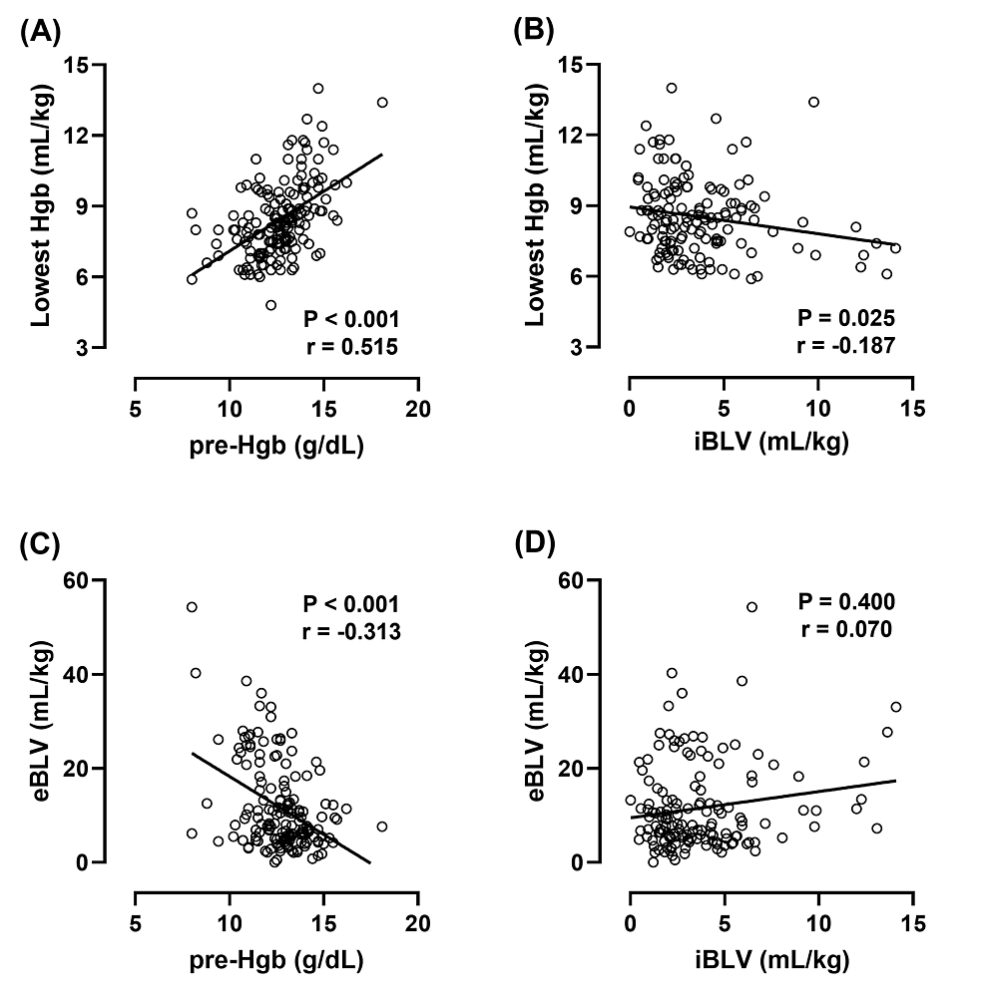
Effects of the preoperative Hgb level and iBLV on postoperative blood loss Preoperative Hgb levels are significantly associated with decreased lowest Hgb levels on POD 3 (A, B) and increased eBLV (C, D). Correlations were evaluated using Spearman's correlation coefficient. The P-values and correlation coefficients (r) are shown in each figure. Pre-Hgb: preoperative hemoglobin; eBLV: estimated blood loss volume per body weight; POD 3: postoperative day 3; Pre: preoperative day; iBLV: intraoperative blood loss volume per body weight

Decreasing preoperative Hgb levels and increasing iBLV were significantly associated with decreasing postoperative Hgb levels (Figures 3A, 3B). In contrast, lower preoperative Hgb levels were significantly associated with higher eBLV, but no significant correlation was found between iBLV and eBLV (Figures 3C, 3D).

Association of preoperative Hgb levels and iBLV with prolonged coagulation parameters in edoxaban thromboprophylaxis

**Figure 4:**
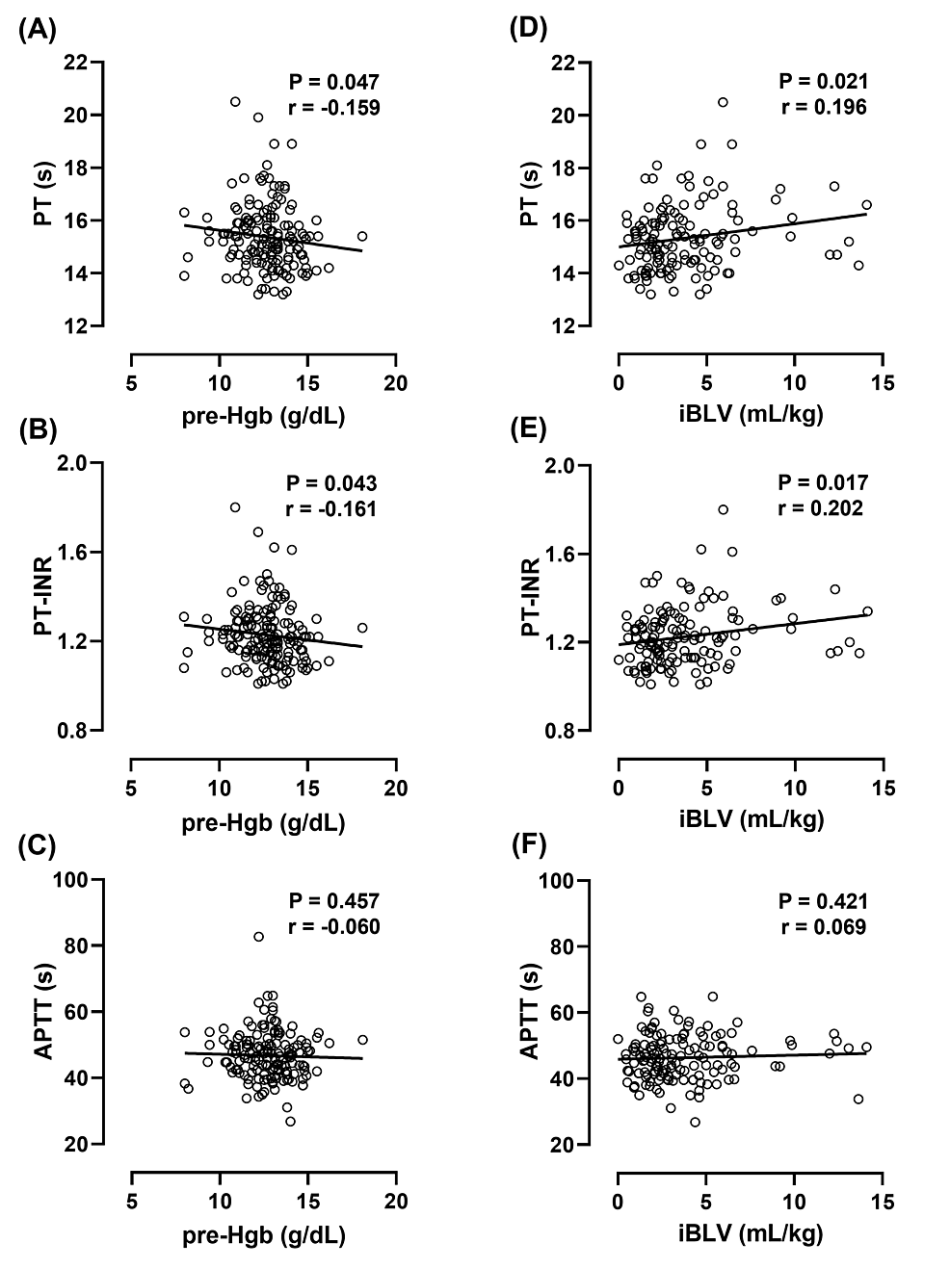
Association between lower preoperative Hgb levels or intraoperative blood loss and prolonged coagulation parameters during edoxaban thromboprophylaxis PT and PT-INR on POD3 are significantly correlated with preoperative anemia (A, B) and intraoperative blood loss (D, E). Furthermore, none of the factors is associated with APTT (C, F). Correlations were evaluated using Spearman's correlation coefficient. The P-values and correlation coefficients (r) are shown in each figure. iBLV: intraoperative blood loss volume per body weight; pre-Hgb: preoperative hemoglobin; pre-OP: preoperative evaluation; POD3: postoperative day 3; PT: prothrombin time; PT-INR: international standardized ratio of prothrombin time; APTT: activated partial thromboplastin time

PT, PT-INR, and APTT were assessed to determine the correlation between preoperative Hgb levels and iBLV (Figure 4). Lower preoperative Hgb levels and higher iBLV were significantly associated with prolonged PT and PT-INR but not APTT during edoxaban thromboprophylaxis (Figure 4).

Association of preoperative Hgb levels and iBLV with preoperative renal function

**Figure 5:**
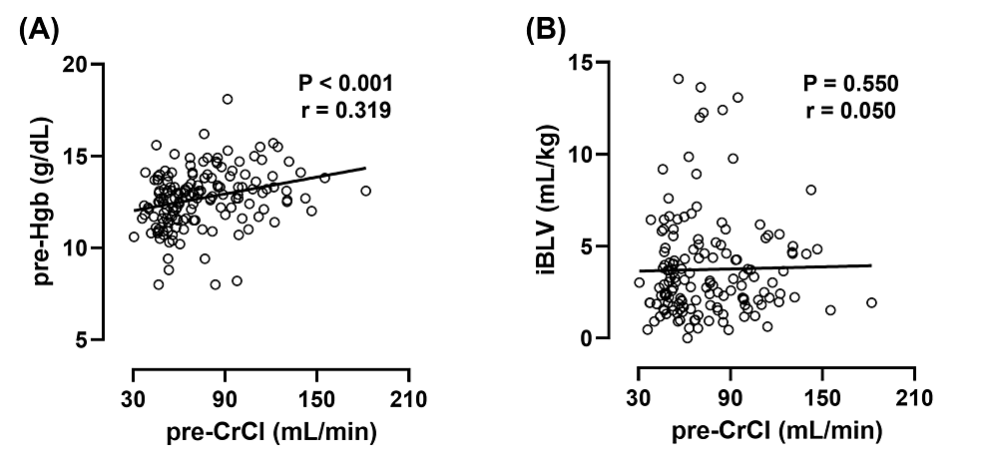
Association between preoperative renal function and preoperative Hgb or iBLV Preoperative Hgb levels were significantly correlated with preoperative creatinine clearance (A). However, iBLV was not significantly correlated with preoperative creatinine clearance (B). Correlations were evaluated using Spearman's correlation coefficient. The P-values and correlation coefficients (r) are shown in each figure. iBLV: intraoperative blood loss volume per body weight; pre-Hgb: preoperative hemoglobin; pre-CrCl: preoperative creatinine clearance

We previously reported the importance of considering impaired preoperative renal function as a risk factor for anemia during edoxaban treatment [7]. Therefore, the relationships between preoperative Hgb values or iBLV and preoperative renal function were evaluated (Figure 5). Preoperative Hgb values were positively correlated with preoperative renal function (P<0.001). However, iBLV was not associated with preoperative renal function.

## Discussion

In this study, we found that lower preoperative Hgb levels and more intraoperative blood loss require attention as risk factors for patients requiring blood transfusion because of worsening anemia during edoxaban thromboprophylaxis after orthopedic surgery. Although these risk factors for transfusion after TKA/THA are well known, this study is the first to show that one or both of these factors against edoxaban thromboprophylaxis are potentially related to a rise in the frequency of blood transfusions by increasing the postoperative PT and PT-INR, decreasing the lowest postoperative Hgb level, and/or increasing the estimated postoperative blood loss.

Preoperative anemia is a common issue in patients who undergo orthopedic surgery and has been associated with an increased risk of transfusion [13]. Furthermore, blood transfusions after orthopedic surgery are associated with increased mortality, infection rate, and length of hospital stay [14]. In this study, patients with lower preoperative Hgb levels had an increased estimated postoperative blood loss during edoxaban treatment after orthopedic surgery. Additionally, bleeding-related events and transfusions were significantly more frequent in patients with lower preoperative Hgb levels. Other researchers have also reported that a low baseline Hgb level is a significant risk factor for bleeding in patients receiving postoperative edoxaban, and the results of this study showed similar characteristics [15].

Intraoperative blood loss was also a risk factor for an increased frequency of blood transfusions, second only to low preoperative Hgb levels. A higher iBLV does not seem to be a risk factor strong enough to affect the increase of eBLV, compared to low preoperative Hgb levels. However, both factors appeared to be significantly associated with the degree of lower postoperative Hgb levels. Therefore, patients scheduled to receive postoperative edoxaban thromboprophylaxis may also require treatment for preoperative anemia and careful intraoperative hemostasis management.

Furthermore, the prolongation of coagulation parameters by edoxaban administration has been reported to depend on its blood concentration [16]. This study demonstrated that preoperative Hgb levels and iBLV were correlated with prolonged coagulation parameters, particularly PT and PT-INR. Furthermore, prolonged coagulation parameters have been associated with a higher eBLV [6]. Therefore, a higher eBLV, especially due to low preoperative Hgb levels, may be associated with prolonged coagulation parameters with edoxaban thromboprophylaxis. However, the detailed mechanism underlying the correlation between preoperative Hgb levels or iBLV and coagulation parameters is poorly understood.

In contrast, lower preoperative Hgb levels were significantly associated with decreased preoperative renal function. A reduction in renal function may be a critical risk factor for anemia accompanied by increased coagulation parameters due to edoxaban thromboprophylaxis [7]. Additionally, preoperative renal function impairment was found to be a critical factor in decreasing Hgb levels among patients experiencing postoperative anemia during edoxaban thromboprophylaxis after orthopedic surgery (Tables 5-6 in Appendices). Therefore, lower preoperative renal function might be related to the significant association between preoperative Hgb levels and postoperative coagulation parameters. These results indicate that a decrease in Hgb levels associated with a preoperative age-related decline in renal function is expected to correlate with an increased risk of postoperative anemia during edoxaban thromboprophylaxis. Therefore, in TKA and THA - whose predominant patient population is older than the general one - both anemia and decreased renal function should be considered before initiating edoxaban thromboprophylaxis. Furthermore, these patients should be vigilant for the risk of anemia requiring transfusion due to the excessive prolongation of coagulation parameters during postoperative edoxaban thromboprophylaxis.

This study has some limitations. First, this was an observational retrospective study conducted at a single center. In addition to decreased renal function and preoperative anemia, several other factors may affect postoperative blood loss during edoxaban thromboprophylaxis. Second, missing data were not statistically compensated for and excluded from the analysis. Finally, the retrospective nature of this study may have introduced bias in the results. To address the effects of regional and institutional differences, patient population biases, and missing data, more accurate multicenter studies and randomized controlled trials involving patients receiving postoperative edoxaban antithrombotic prophylaxis are needed.

Furthermore, there was a discrepancy between the period during which the patient data were collected for this study and when the results were compiled. We have been studying the relationship between edoxaban thromboprophylaxis after TKA/THA and postoperative anemia since 2012 [5-7]. During the study period, a report in 2020 revealed low baseline Hgb levels as a new risk factor for bleeding-related events, including transfusion during edoxaban administration [15]. As our study had already identified an increasing trend in postoperative blood transfusions with edoxaban thromboprophylaxis, we assessed the possibility of a new risk factor for patients with easily worsening anemia that would require blood transfusions. This resulted in the long-term difference between the periods where the data were collected and when the results were reported in this study.

## Conclusions

Our study showed that preoperative Hgb reduction and elevated iBLV may be associated with a further increase in the transfusion risk by prolonging coagulation parameters, including PT and PT-INR, during edoxaban thromboprophylaxis. Therefore, physicians treating patients with not only a decline in preoperative renal function but also preoperative anemia and high intraoperative blood loss should be aware of the risk of the exacerbation of anemia during edoxaban thromboprophylaxis administration.

Finally, expanding on the clinical significance of the effect of preoperative anemia and intraoperative blood loss as risk factors for transfusion during edoxaban thromboprophylaxis would provide valuable insights for orthopedic surgeons and healthcare providers managing patients undergoing total knee or hip arthroplasty.

## Appendices

**Table 4:** Reasons for the discontinuation of edoxaban thromboprophylaxis and their frequency VTE: venous thromboembolism

Events	Transfusion (-) N=120	Transfusion (+) N=47	P-value
Hospital discharge or changing hospital (+/-)	11 / 109	2 / 45	0.356
Bleeding-related event (+/-)	1 / 119	4 / 43	0.023
Switching to other anti-thrombogenic drug (+/-)	3 / 117	1 / 46	>0.999
Treatment of VTE (+/-)	2 / 118	1 / 46	>0.999
Others (complications or unknown) (+/-)	1 / 119	2 / 45	0.191

**Table 5:** Characteristics of patients in the groups with or without a reduction in Hgb level of at least 2 g/dL at preoperative assessment Data are expressed as the mean±standard deviation or absolute values. #Patients were excluded if data were missing. TKA: total knee arthroplasty; THA: total hip arthroplasty; CrCl: creatinine clearance; Hgb: hemoglobin; iBLV: intraoperative blood loss volume per body weight; PT: prothrombin time; PT-INR: prothrombin time-international normalized ratio; APTT: activated partial thromboplastin time

Characteristics	Hgb reduction (-) N=117	Hgb reduction (+) N=50	P-value
Sex (male/female)	15 / 102	16 / 34	0.008
Age (years)	72.6 ± 8.4	74.4 ± 10.1	0.076
Surgical method (TKA/THA)	72 / 45	36 / 14	0.220
Height (cm)	151.3 ± 7.0 (n=113^#^)	154.7 ± 9.9 (n=48^#^)	0.044
Body weight (kg)	59.4 ± 12.1 (n=113^#^)	59.2 ± 11.8 (n=48^#^)	0.967
CrCl (mL/min)	77.3 ± 29.1 (n=113^#^)	65.4±24.5 (n=48^#^)	0.004
Hgb (g/dL)	12.5 ± 1.5	13.1 ± 1.8	0.034
iBLV (mL/kg)	3.77 ± 2.61 (n=103^#^)	3.64 ± 3.10 (n=44^#^)	0.339
PT	13.2 ± 0.9	13.4 ± 1.1	0.453
PT-INR	1.01 ± 0.09	1.03 ± 0.12	0.336
APTT	35.4 ± 3.6	35.6 ± 3.5	0.936
Combined anti-thrombogenic drug use (+/-)	17 / 100	14 / 36	0.051
Combined P-glycoprotein inhibitor use (+/-)	2 / 115	4 / 46	0.066

**Table 6:** Univariable and multivariable logistic regression analyses of risk factors for decreasing Hgb levels during edoxaban thromboprophylaxis OR: odds ratio; VIF: variance inflation factor; CI: confidence interval; CrCl: creatinine clearance; Hgb: hemoglobin

	Univariable logistic regression analyses			Multivariable logistic regression analysis			
Factors	OR	95% CI	P-value	OR	95% CI	VIF	P-value
Sex (male/female)	3.200	1.431–7.224	0.005	1.929	0.571–6.581	1.802	0.287
Height	1.055	1.011–1.102	0.015	1.050	0.983–1.125	2.122	0.151
CrCl	0.983	0.968–0.996	0.017	0.969	0.951–0.986	1.180	0.001
Hgb	1.271	1.021–1.607	0.037	1.298	0.999–1.729	1.193	0.060
